# Evaluation of cytokine expression and circulating immune cell subsets as potential parameters of acute radiation toxicity in prostate cancer patients

**DOI:** 10.1038/s41598-020-75812-0

**Published:** 2020-11-04

**Authors:** Tatjana P. Stanojković, Ivana Z. Matić, Nina Petrović, Vesna Stanković, Katarina Kopčalić, Irina Besu, Marija Đorđić Crnogorac, Emina Mališić, Katarina Mirjačić-Martinović, Ana Vuletić, Zoran Bukumirić, Željko Žižak, Marlon Veldwijk, Carsten Herskind, Marina Nikitović

**Affiliations:** 1 Institute of Oncology and Radiology of Serbia, Belgrade, Serbia; 2 “VINČA” Institute of Nuclear Sciences-National Institute of the Republic of Serbia, University of Belgrade, Belgrade, Serbia; 3 Institute for Medical Statistics and Informatics, School of Medicine, University of Belgrade, Belgrade, Serbia; 4 Department of Radiation Oncology, University Medical Center Mannheim, Medical Faculty Mannheim, University of Heidelberg, Mannheim, Germany; 5 School of Medicine, University of Belgrade, Belgrade, Serbia

**Keywords:** Biomarkers, Oncology

## Abstract

One of the challenges of radiation oncology in the era of personalized medicine is identification of biomarkers associated with individual radiosensitivity. The aim of research was to evaluate the possible clinical value of the associations between clinical, physical, and biological factors, and risk for development of acute radiotoxicity in patients with prostate cancer. The study involved forty four patients treated with three-dimensional conformal radiotherapy. The concentrations of IL-1β, IL-2, IL-6, IFN-γ and TGF-β1 were assessed before radiotherapy, after 5th, 15th and 25th radiotherapy fractions, at the end, and 1 month after the end of radiotherapy. Cytokine gene expression was determined in peripheral blood mononuclear cells. The univariate analysis of circulating cytokine levels during radiotherapy showed that increased serum concentrations of IL-6 were significantly associated with higher grade of acute genitourinary toxicity. The multivariate analysis demonstrated that increased level of IL-6 during the radiotherapy was significantly associated with higher grade of acute genitourinary toxicity across treatment. *TGF-β* expression levels significantly decreased during course of radiotherapy. Research indicates that changes in circulating cytokine levels might be important parameter of radiotoxicity in patients with prostate cancer. These findings suggest that future studies based on multi-parameter examination are necessary for prediction of individual radiosensitivity.

## Introduction

In the era of personalized medicine, radiotherapy aims to permanently control a primary malignant tumor and regional lymph node metastases and thus avoiding normal tissue toxicity. Between 5% and 10% of cancer patients treated with radiotherapy develop severe side effects which may influence the response to radiotherapy and patients’ quality of life^1,2^. Although efforts are constantly made to improve radiation therapy techniques, dose delivery, and optimization of treatment regimens further understanding of individual biological differences in patients is needed for development of personalized radiotherapy^3^. There is a considerable interest in identifying biomarkers associated with patient's individual radiosensitivity. According to the literature data Rubin et al. were among the pioneers in the field of radiation-induced toxicity in association with cytokine level^4^. Cytokine expression is associated with radiation-related tissue damage and inflammation, and may serve as an indicator of cell and tissue toxicity during prostate cancer radiotherapy^5,6^. Exposure to radiation initiates a programmed cellular response to promote tissue repair, which involves the induction and regulation of proinflammatory cytokines. Inflammation is thought to play a key role in the response of malignant tumors to radiotherapy. Previously published studies indicate an increase in circulating levels of proinflammatory cytokines during radiotherapy, such as IFN-ɣ, IL-6, TNF-α, and IL-4^7,8^. Thus Stenmark et al. suggested that assessment of cytokines IL-8 and TGF-β in combination with dose parameters may be useful for prediction of radiation–induced lung toxicity^2^. Furthermore, it has been shown that there is a difference in the level of cytokines in patients with prostate cancer compared with patients with benign lesions and healthy individuals, as well as changes after radiotherapy and/or androgen therapy^3,9^. Changes in the level of inflammatory biomarkers have also been shown to be positively correlated with fatigue symptoms, as well as with acute gastrointestinal and genitourinary toxicity^5,10^. Radiotherapy in patients with prostate cancer causes inflammation leading to an increase in the circulating levels of IL-6, IL-8, TNF-α and TGF-β^8^. The study by Christensen et al.^5^ showed significant increases in serum levels of IFN-γ and IL-6 during radiotherapy in patients with prostate cancer. The elevated levels of IL-1 and IL-2 were associated with the risk of genitourinary and gastrointestinal radiotoxicity development^5^.

Changes in levels of proinflammatory cytokines have been associated not only with radiation-induced toxicity, but also with tumor response to radiotherapy. It has been found that an increase in IL-6 levels contributes to more aggressive tumor growth and resistance to treatment. The therapeutic modulation of IL-6 could increase the sensitivity of malignant tumors to radiotherapy^11^. An increase in the percentage of regulatory T lymphocytes (Tregs) occurs in patients receiving radiotherapy, in various tumors, which is associated with poor therapeutic response^11,12^. The radiotherapy response was inversely correlated with cytokine expression (TGF-β) and the level of Tregs in a mouse model^13^. Likewise, surgery has been shown to lead to increased expression of cytokines similar to those found in the inflammatory response of gastrointestinal mucosa after radiotherapy^5,14–16^. However, the complex role of proinflammatory cytokines in radiation therapy-related toxicity has not yet been completely elucidated.

The aim of the present study was to examine the potential clinical significance of the associations between clinical, physical, and multiple biological parameters from three different levels, which derived from the previous study which investigated association only between clinico-pathological and physical parameters and potential risk for development of acute radiotoxicity^17^.

The longitudinal cytokine profiles in serum and gene expression levels in peripheral blood mononuclear cells for the prediction of individual sensitivity to radiotherapy in patients with prostate cancer in addition, the changes in the percentage of peripheral white blood cell subpopulations in these patients throughout the course of radiation therapy were assessed, as well. This integrated multi-level approach provides a better insight into biological effects of fractionated radiotherapy in the terms of more precise prediction of acute radiotoxicity. The examined six time points represent the resultant of cumulative effect of radiation during the therapy.

## Results

### Patients characteristics and radiation-induced toxicity

**Figure 1:**
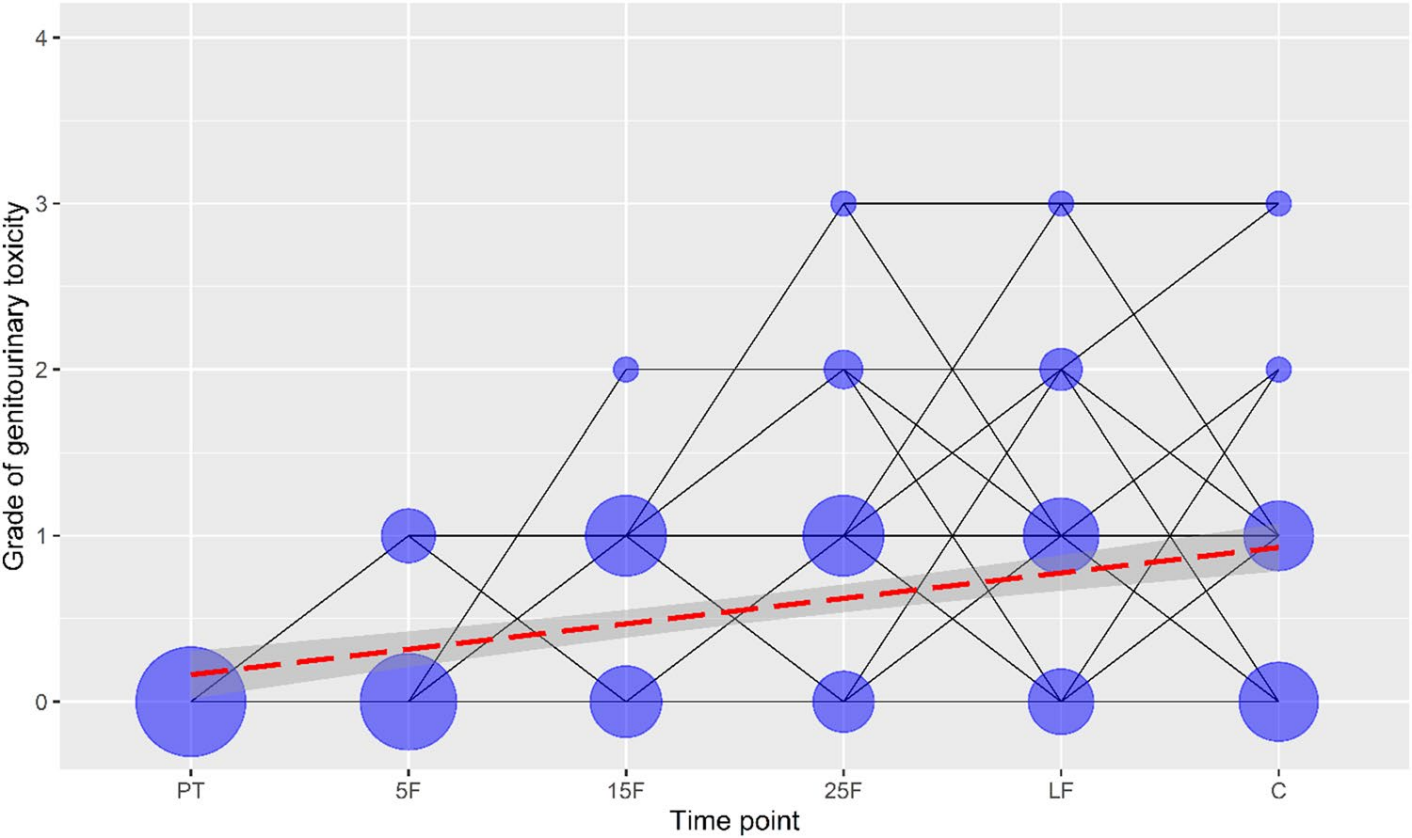
Changes in acute genitourinary toxicity grades in specific time points: PT-pre-treatment, 5F-after the 5th of radiotherapy, 15F- after the 15th fraction of radiotherapy, 25F-after the 25th fraction of radiotherapy, LF-at the last fraction of radiotherapy, and C-at the first control examination. Lines represent acute genitourinary toxicity grade changes in particular time points for each patient. Circle area is proportional to the number of patients. In the pre-treatment point it is 100%. On each time point, sum of circle area is 100%.

The characteristics of the study cohort are shown in Table 1. Patient age ranged from 52 to 81 years, with a mean age of 69.2 years ± 7.1. Among examined patients 10 patients (21.3%) had diabetes mellitus type 2 (non-insulin dependent), 23 patients (48.9%) were active smokers during radiotherapy, 19 patients (40.4%) had chronic hypertension. Median Gleason score (GS) was 7 (min 6, max 9). Low, intermediate and high risk prostate cancer had 3 patients (8.3%), 14 patients (38.9%) and 19 patients (52.8%) respectively. For the whole group of patients, the initial level of prostate-specific antigen (PSA) in the blood before making clinical diagnosis ranged from 4.63 ng/mL to 57 ng/mL (median value was 14.30 ng/mL). Definitive radiotherapy was performed in 21 patients (47.7%). Postoperative radiotherapy was performed in 23 patients (52.3%); in 6/23 patients radiotherapy was adjuvant, while in 17/23 patients radiotherapy was salvage. Residual microscopic disease after radical prostatectomy was present in 9 out of 23 patients. After the 25th fraction of radiotherapy, grade 1 was the most common acute genitourinary radiotoxicity with a smaller fraction of patients showing grade 2 and 3 (Fig. 1).

**Table 1:** Patient, clinical, tumor, and treatment characteristics.

Variable	Description	Univariate analysis*	
		b	*p*
**Mean age ± standard deviation**	69.2 ± 7.1	0.020	0.152
**Diabetes mellitus type 2**			
Yes	10 (21.3%)	0.030	0.849
No	34		
**Smoking status**			
Active/former	23 (48.9%)	− 0.176	0.204
Non-smokers			
**Chronic hypertension**			
Yes	19 (40.4%)	0.018	0.895
No			
**Tumor volume in % of tissue sample, median, min, max**	40 (12–80)	0.004	0.343
**Gleason score, median, min, max**	7 (6–9)	− 0.016	0.857
**Risk group**			
1 (low)	3 (8.3%)	0.131	0.231
2 (intermediate)	14 (38.9%)		
3 (high)	19 (52.8%)		
**R1 resection**	9 (75.0%)	− 0.438	0.147
**Type of radiotherapy**			
Postoperative	23 (52.3%)	0.003	0.815
Definitive	21 (47.7%)		

*Multilevel ordinal regression models with the degree of acute genitourinary toxicity as the dependent variable, adjusted for the observation and the type of treatment. Depending on the type of variables and the normality of distribution, data are shown as n (%), as ± sd or median (min–max).

### Changes in serum cytokine levels

To investigate whether changes in cytokine profiles correlate with radiation-induced acute genitourinary toxicity, the concentrations of IL-1β, IL-2, IL-6, IFN-γ and TGF-β1 were measured in serum of patients with prostate cancer undergoing radiotherapy before treatment initiation, after 5th, 15th and 25th radiotherapy fractions, at the end of radiotherapy after last fraction, and 1 month after the end of radiotherapy.

The univariate analysis of circulating cytokine levels showed that the concentrations of IL-2, IFN-γ, and TGF-β1 in sera of patients with prostate cancer tended to increase, but not statistically significant during course of radiotherapy (Table 2). Increased serum concentrations of IL-6 were significantly associated with higher grade of acute genitourinary toxicity (b = 0.018; *p* = 0.039). The trend for an association between increased concentrations of IL-2 and higher grade of acute genitourinary toxicity (b = 0.018; *p* = 0.081) was observed.

**Table 2:** Univariate analysis of cytokine levels in patients with prostate cancer during course of radiotherapy.

Concentration [pg/mL]	Pre-treatment	After 5th fraction	After 15th fraction	After 25th fraction	After last fraction	After 1 month	b	*p*
**Univariate analysis**								
IL-1β	1.2 (0.1–4.1)	1.0 (0.2–4.3)	1.3 (0.3–4.2)	1.3 (0.3–4.3)	1.2 (0.3–4.5)	1.2 (0.2–4.8)	0.018	0.712
IL-2	1.9 (0.2–27.9)	1.8 (0.2–17.8)	1.6 (0.3–32.2)	2.1 (0.3–26.1)	1.8 (0.2–36.4)	1.8 (0.2–18.8)	0.018	0.081
IL-6	2.6 (0.3–14.3)	2.5 (0.2–29.3)	2.4 (0.2–40.0)	2.6 (0.2–33.8)	1.9 (0–58.6)	2.5 (0.1–17.3)	0.018	**0.039**
IFN-γ	8.1 (2.2–96.3)	9.8 (1.3–52.1)	8.8 (1.3–78.0)	8.3 (1.8–90.5)	8.4 (1.3–107.7)	6.8 (1.9–52.9)	0.004	0.138
TGF-β1	42.7 (4.3–155.0)	43.1 (4.2–91.0)	46.3 (4.9–149.7)	44.6 (5.9–150.3)	45.6 (1.5–120.7)	41 (2.8–193.5)	0.001	0.516

*Multilevel ordinal regression models with the degree of acute genitourinary toxicity as the dependent variable, adjusted for the time point and the type of treatment. Depending on the type of variables and the normality of distribution, data are shown as median (min–max).

Multivariate multilevel ordinal regression analysis was used to determine whether changes in percentage of circulating lymphocytes and concentrations of IL-2 and IL-6 levels in sera of patients with prostate cancer treated with radiotherapy predicted changes in acute genitourinary toxicity over time. This analysis showed that decreased percentage of lymphocytes during time was significantly associated with higher grade of acute genitourinary toxicity (b = − 0.013, *p* = 0.025, Table 3). Considering the changes in cytokine profiles, increased level of IL-6 during the course of radiotherapy was significantly associated with higher grade of acute genitourinary toxicity across treatment (b = 0.017, *p* = 0.046, Table 3). In addition, the increased level of IL-2 was associated with higher grade of acute genitourinary toxicity across treatment, although this association did not reach statistical significance (b = 0.020, *p* = 0.062, Table 3).

**Table 3:** Multivariate multilevel ordinal regression model of percentage of lymphocytes and concentrations of cytokines in patients with prostate cancer.

Parameter	Multivariate analysis*	
	b	*p*
Percentage of lymphocytes	− 0.013	**0.025**
IL-2	0.020	0.062
IL-6	0.017	**0.046**

*Multilevel ordinal regression model with the degree of acute genitourinary toxicity as the dependent variable, adjusted for the time point and the type of treatment.

This finding for IL-2 might be attributed to the relatively small number of tested patients and/or to low toxicity grades during the course of radiotherapy observed in the group of analyzed patients. Thus seven patients had toxicity grades 2 and 3 after the 25th radiotherapy fraction, while 8 patients had toxicity grades 2 and 3 at the end of radiation therapy.

The possible differences in temporal profiles of circulating cytokines and their association with acute genitourinary toxicity were examined between patients treated with definitive radiotherapy who had intact prostate and patients treated with postoperative radiotherapy who had prostatectomy. According to the mixed effect model that analyses each time point for each individual, levels of IL-6 and IFN-γ were significantly higher in the subgroup of patients treated with definitive radiotherapy than in the subgroup of patients treated with postoperative radiotherapy (*p* = 0.001 and *p* = 0.020, respectively, Supplementary Table 1).

The univariate analysis of the percentage of leukocyte or lymphocyte subpopulations in patients before radiotherapy, during the course of radiotherapy, and one month after end of radiotherapy is presented in Supplementary Table 2.

### Cytokine gene expression levels in peripheral blood mononuclear cells (PBMCs)

**Figure 2:**
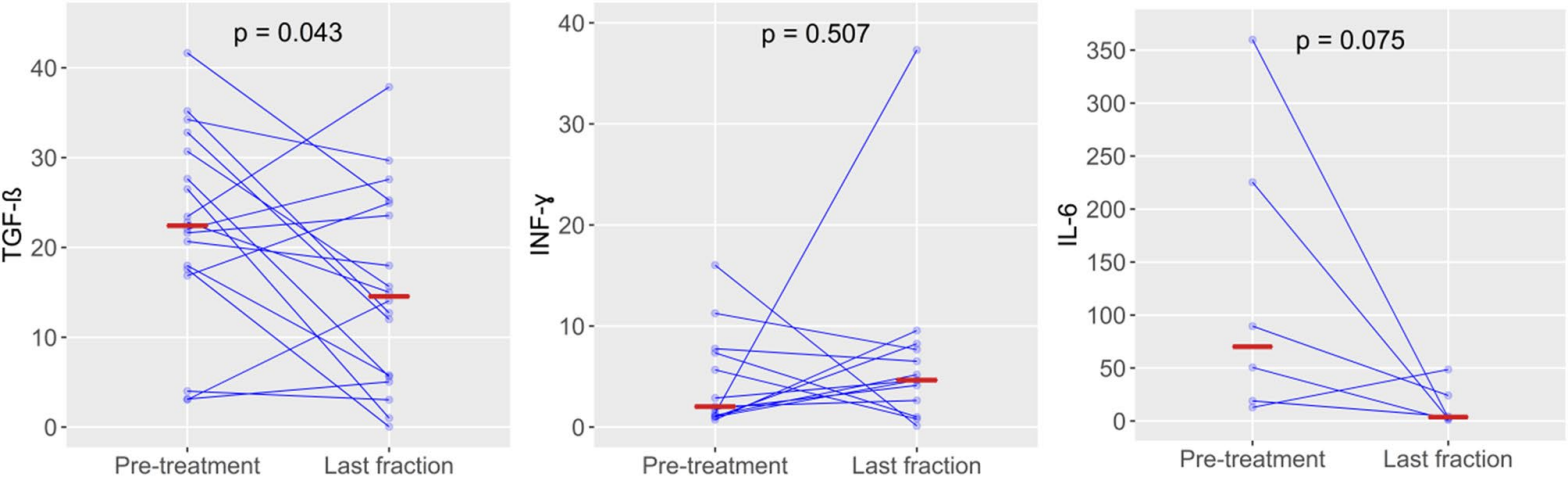
Cytokine (*TGF-β*, *INF-ɣ*, and *IL-6*) gene expression time profile at pre-treatment time point and last fraction of radiotherapy-pairwise comparison.

Firstly, we compared pre-treatment cytokine gene expression levels with levels measured after the last fraction of radiotherapy. *TGF-β* gene expression levels significantly decreased during radiotherapy (*p* = 0.043, Wilcoxon Signed Ranks Test, Fig. 2, Table 4) while *IL-6* levels showed a non-significant trend towards lower (*p* = 0.075, Wilcoxon Signed Ranks Test, Table 4, Fig. 2).

**Table 4:** *TGF-β*, *INF-ɣ*, *IL-6* gene expression profile at pre-treatment time point and after the last fraction of radiation therapy.

Gene	Time point	N	Median, minimum, maximum	*p* value
*TGF-β*	Pre-treatment	18	22.4 (3.0–41.7)	**0.043**
	Last fraction	18	14.5 (0.05–37.9)	
*INF-ɣ*	Pre-treatment	13	2.0 (0.7–16.0)	0.507
	Last fraction	13	4.6 (0.1–37.3)	
*IL-6*	Pre-treatment	6	70.1 (12.8–359.8)	0.075
	Last fraction	6	3.7 (1.0–48.5)	

N-number of patients. Wilcoxon’s test, pairwise comparison *p* values equal or less than 0.05 were considered significant, and *p* values lower than 0.1 were considered as statistical trend.

Secondly, we examined correlations between gene expression measured in PBMCs and protein levels measured in sera of matched patients, and detected no significant correlations in none of the compared groups (Table 5).

**Table 5:** Correlations between gene and protein levels at pre-treatment point and after the last fraction of radiotherapy.

Variable 1	Variable 2	N	Time point	Correlation coefficient	*p* value
TGF-β protein	*TGF-β* gene	22	Pre-treatment	rho = 0.127	0.573
		25	Last fraction	rho = − 0.135	0.519
INF-ɣ protein	*INF-ɣ* gene	14	Pre-treatment	rho = 0.270	0.350
		22	Last fraction	rho = − 0.343	0.118
IL-6 protein	*IL-6* gene	9	Pre-treatment	rho = − 0.400	0.286
		14	Last fraction	rho = − 0.150	0.610

N-number of patients. Spearman’s correlational test-p values equal or less than 0.05 were considered significant, and *p* values lower than 0.1 were considered as statistical trend.

### Correlations between lymphocyte subsets and gene expression levels of cytokines

Correlation between immunophenotype (lymphocyte subsets) and gene expression levels of *IL-6*, *IFN-γ*, and *TGF-β* at pre-treatment time point and after the last fraction of radiotherapy showed a significant negative correlation between *IL-6* and Foxp3^+^ (rho = − 0.580, *p* = 0.048, Table 6) or CD4^+^CD25^+^Foxp3^+^ (rho = − 0.578, *p* = 0.049, Table 6) after the last fraction of radiotherapy. For the level of *IFN-γ* gene expression, a significant negative correlation was found with the percentage of Foxp3^+^CD4^+^CD25^+^ cells in PBL at pre-treatment time point and after the last fraction of radiotherapy (rho = 0.491, *p* = 0.053; rho = − 0.660, *p* = 0.002, respectively, Table 6). After the last fraction of radiotherapy the level of *IFN-γ* gene transcription shows significant negative correlation with the percentage of Foxp3^+^ (rho = − 0.495, *p* = 0.027, Table 6) and CD4^+^Foxp3^+^ (rho = − 0.685, *p* < 0.001, Table 6) cells in PBL. For the level of expression of *TGF-β* gene we found significant positive correlation with the mean fluorescence intensity (MFI) of Foxp3 transcription factor in PBL after the last fraction of radiotherapy (rho = 0.463, *p* = 0.026, Table 6).

**Table 6:** Correlation of percentage of lymphocyte subset and gene expression level.

Percentage of lymphocyte subset	Gene expression level	N	Time point	Correlation coefficient	*p* value
Foxp3	*IL-6*	9	Pre-treatment	rho = − 0.317	0.406
Foxp3	*IL-6*	12	Last fraction	rho = − 0.580	**0.048**
CD4^+^CD25^+^Foxp3	*IL-6*	9	Pre-treatment	rho = − 0.326	0.391
CD4^+^CD25^+^Foxp3	*IL-6*	12	Last fraction	rho = − 0.578	**0.049**
CD4^+^CD25^+^Foxp3	*INF-ɣ*	16	Pre-treatment	rho = − 0.591	**0.007**
CD4^+^CD25^+^Foxp3	*INF-ɣ*	20	Last fraction	rho = − 0.660	**0.002**
Foxp3	*INF-ɣ*	16	Pre-treatment	rho = 0.181	0.502
Foxp3	*INF-ɣ*	20	Last fraction	rho = − 0.495	**0.027**
CD4^+^Foxp3	*INF-ɣ*	16	Pre-treatment	rho = − 0.069	0.799
CD4^+^Foxp3	*INF-ɣ*	20	Last fraction	rho = − 0.685	** < 0.001**
Foxp3 MFI*	*TGF-β*	21	Pre-treatment	rho = 0.019	0.935
Foxp3 MFI	*TGF-β*	23	Last fraction	rho = 0.463	**0.026**

*MFI-Mean Fluorescence Intensity.

Correlation between percentage of lymphocyte subpopulation, MFI and relative expression levels of *IL-6*, *INF-ɣ*, and *TGF-β*. *p* values less than 0.05 were considered as statistically significant.

## Discussion

Many studies indicate that the assessment of temporal profiles of circulating cytokine levels in patients undergoing radiotherapy could be a valuable approach for elucidation of their involvement in development of acute and late toxicity caused by radiological treatment as well as for monitoring radiotherapy response. The longitudinal examination of circulating cytokine profiles in our group of patients with prostate cancer treated with conventionally fractionated conformal radiotherapy showed that increased levels of IL-2 and IL-6 during the treatment course were associated with higher grade of acute genitourinary toxicity. Our finding indicates that circulating IL-6 may be associated with acute radiation toxicity.

The results from a similar longitudinal study of cytokine expression patterns suggested that serum levels of IL-1, IL-2, IL-6 and IFN-γ in patients with prostate cancer during intensity-modulated radiotherapy may be predictive for acute radiation-induced normal tissue toxicity^5^. The changes in IL-2, IL-6 and IFN-γ over baseline were associated with increased gastrointestinal toxicity, while the changes in IL-1 and IFN-γ were associated with increased genitourinary toxicity^5^. Although Christensen et al.^5^ found that circulating IL-6 was significantly elevated in patients with prostate cancer during radiotherapy, this increase of IL-6 level was not associated with acute genitourinary toxicity as it was observed in our study. Differences between those two studies might be the consequence of the fact that they have used IMRT, while we used 3D conformal radiotherapy, and because of individual differences among patients and observed time points. The results of prospective, longitudinal, phase II study showed that the serum levels of IL-6 tended to increase in patients with early stage prostate cancer during external beam radiotherapy^18^. The observed differences in temporal patterns of cytokine expression and their relationship with acute toxicity in prostate cancer patients between different studies might be attributed to differences in radiotherapy modalities, dose and volume of the irradiated tissue, in addition to differences in individual patient’s response to radiotherapy. It has been suggested that various biological factors may affect radiation – induced inflammatory response and cytokine signatures, such as genetic and epigenetic factors, pathohistological and molecular characteristics of the malignant tumors, tumor microenvironment, patient’s general health condition in addition to radiation therapy dose and volume of the irradiated tissue^6,19,20^. The cytokine IL-6 has been suggested as one of the most promising immunological markers that might be useful as additional factor for prediction of radiation-induced normal tissue toxicity^6,19,21^, not only for prostate cancer, but also for other types of cancer. The research conducted by Siva et al.^22^ reported that the early alterations in plasma IL-6 concentrations had been related with higher grade lung toxicity in patients with non-small cell lung cancer treated with radiotherapy^22^.

Evaluation of possible prostate-cancer specific cytokine signatures in our group of patients showed significantly higher circulating levels of IL-6 and IFN-γ in subgroup of patients receiving definitive radiotherapy in comparison with subgroup of patients receiving postoperative radiotherapy, which might be the consequence of higher maximal dose (72 Gy definitive radiotherapy group, and 66 Gy dose for postoperative and salvage radiotherapy group), or the consequences of irradiated volumes, that differed among groups, or the result of interplay between various factors such as presence of prostate and irradiation.

This finding for IL-6 was expected since prostate cancer cells produce this pro-inflammatory cytokine involved in the inflammatory response to ionizing radiation^6,23^. Tazaki et al.^24^ reported increased serum level of IFN-γ in patients with prostate carcinoma^24^.

There is growing evidence that PBMCs are capable of releasing a biologically active secretome^25^. It is a well-known fact that the first lines of response to irradiation are cells of the immune system. Helper T lymphocytes CD4^+^ T (Th) are extremely radiosensitive cell population^26^. TGF-β regulates differentiation, immune response, and development of T cells and regulates formation of Foxp3^+^ regulatory T and inflammatory T helper Th17 cells^27^.

In our research we have measured systemic cytokine gene expression profile in PBMC of the same patients that we measured sera levels. We did not find any significant correlation between TGF-β, INF-ɣ, and IL-6 gene-protein levels from PBMCs and serum, respectively, probably due to a great complexity of molecular interactions of the cytokine secretome, including miRNA-mRNA interaction and two models-translational repression, RNA decay, or combined effect^28^. Secondly, different cell subtypes such as endothelial cells, fibroblasts, etc. produce cytokines, so sera contain the complete cytokine secretome, unlike PBMCs.

According to our results, TGF-β and IL-6 levels in PBMCs decreased during the course of radiotherapy compared with baseline. Stenmark et al.^2^ showed radiation-induced increase of plasma levels of TGF-β and IL-6 in lung cancer patients. Bouquet et al. have shown that TGF-β inhibition increases radiosensitivity of breast tumor cells^29^. IL-6 level decrease is associated with increased sensitivity to radiotherapy^29^ and with radiotoxicity. Lowering of TGF-β might be associated with increased radiosensitivity after the last fraction of radiotherapy.

Lymphocytes are generally regarded as radiosensitive and indeed the overall percentage of circulating lymphocytes in peripheral blood decreased during the course of radiotherapy. However, in patients with prostate cancer undergoing radiotherapy before treatment initiation, there was no significant effect on the percentage of CD4^+^ and CD8^+^ T lymphocytes, Tregs, NK or NKT cell subsets at the end of radiotherapy. Numerous studies have shown that Tregs contribute to development and progression of various malignant tumors. However, it has been shown that radiotherapy inhibits apoptosis of Tregs and thereby increases the production of Tregs and also the recruitment of Tregs to local tumor microenvironment^30^. IL-6 is a pleiotropic cytokine involved in the regulation of the immune response, inflammation and hematopoeisis. IL-6 can be detected in the serum, although baseline levels are low in the absence of inflammation. IL-6 is also involved in regulation of the balance between two T cell subsets: Tregs and Th17, which have opposing functions in the control of inflammation^31^. In this study there is a negative correlation between the level of *IL-6* gene expression and the percentage of Tregs in PB in patients with prostate cancer which is the most prominent after last fraction of radiotherapy. These findings are in agreement with well-established role of IL-6 in downregulation of the expression of Foxp3 transcription factor that represents the lineage specific marker of Tregs and therefore IL-6 inhibits differentiation of Tregs^32,33^. Regulatory T cells (Tregs) have the role to suppress immune response maintaining homeostasis and self-tolerance. Therefore, by various mechanisms these cells suppress the immunity to infection and tumors. In this study we found the negative correlation between the percentage of Tregs and *IFN-γ* gene level, especially after last fraction of radiotherapy. It has been shown that Tregs selectively reduce the transcriptional level of IFN-γ without blocking the expression of its Tbet transcription factor. In addition, Treg-mediated blockade of IFN-γ is dependent on Treg-derived IL-10 immunosuppressive cytokine^34^. Tregs are the most important producers of TGF-β. In this study there is significant positive correlation between the MFI of Foxp3 transcription factor in PBL and *TGF-β* gene transcription after the last fraction of radiotherapy. This cytokine released after radiotherapy displays protumorogenic and prometastatic role in some tumors^35^.

## Conclusion

Our study shows that changes in circulating cytokine levels during the course of radiation therapy rather than absolute levels might be important parameters of radiotoxicity. The levels of IL-6 significantly changes during radiotherapy, and is associated with the time of measurement and with the presence of prostate and radiotherapy type, IL-2 levels change during the course of radiotherapy independently of the presence of prostate, while changes in INF-ɣ depend on the presence of prostate and radiotherapy type. Our findings point out that future studies should be designed with more levels of patients subgrouping and stratification according to type of radiation treatment and radiation schedules in order to lower the influence of physical and biological factors. The future of test development for prediction of individual radiosensitivity might be based on determination of cytokine levels changes at specific time points during course of radiotherapy which is only a part of the complex multifactorial puzzle influencing the radiotoxicity.

## Methods

### Patients

Forty four patients who had a histologically confirmed localized or locally advanced prostate cancer (PC) were treated with 3D conformal radiotherapy (3DCRT) at the Institute of Oncology and Radiology of Serbia from January 2016 to August 2017 with definitive (in 21 patients) or postoperative (23 patients) radiotherapy without previous hormonal therapy. All patients had Karnofsky index (KI) ≥ 80. The details of the study were explained to the patients and informed consent forms were signed by the participants. The study protocol was approved by the Ethics Committee of the Institute of Oncology and Radiology of Serbia (approval No3348/1–01). The study was carried out according to the principles of the Declaration of Helsinki.

Acute radiotoxicity was evaluated according to RTOG/EORTC (Acute Radiation Morbidity Scoring Criteria) modified by Peeters^36^, as well in our previous study^17^. According to Peeters and coworkers, side-effects occurring within 120 days from the start of radiotherapy were considered as acute radiation morbidity. Late toxicity was scored from 120 days after the start of treatment any times. All clinical data, and blood samples, were prospectively collected. Serum and PBMC samples were obtained from all patients who were to participate in the studies. The radiation oncologist recorded baseline symptoms and acute symptoms during radiotherapy and at first control (30 days after the end of radiotherapy). Exclusion criteria were as follows: chronic infective diseases, neoadjuvant or concomitant hormonal therapy, the presence of enlarged lymph nodes (N1 stage) detected by imaging methods, the presence of distant metastasis (M1 stage) detected by imaging techniques, Karnofsky index < 80, and previous pelvic irradiation.

### Treatment regimen

3DCRT was performed according to the protocol of the Institute of Oncology and Radiology of Serbia, partly described in our previous published article^17^. Patients treated with definitive radiotherapy received irradiation to different volumes according to the estimated risk of seminal vesicle (SV) involvement, and lymph node involvement according to the Roach formula. Thus patients were allocated to one of the following three groups: (1) prostate-only group (P) if the risk for SV involvement was < 15%; (2) prostate and seminal vesicle group (P + SV), if the risk SV involvement was ≥ 15%; (3) whole pelvic radiotherapy (WPRT) group if the risk for lymph node involvement was ≥ 15%. Clinical target volume (CTV) and Planning target volume (PTV) were used as standardized nomenclature according to International Commission on Radiation Units and Measurements recommendations ICRU 50 and ICRU 62^37,38^. CTV included the whole prostate. CTV1 included whole prostate with entire seminal vesicle. CTV lnn encompassed the lymph node below of bifurcation of *a. iliaca communis*. The planning target volumes (PTV and PTV1) included 10 mm margins around the CTV or CTV1 and CTV lnn except the posterior margin which was reduced to 8 mm. PTV2 and PTV3 included margins around CTV. Margins for PTV2 and PTV3 were reduced to 5 mm except the posterior margin which was further reduced to 0 mm.

Patients were irradiated with a conventional fractionation regime: 2 Gy daily, 5 days weekly.

The prescribed dose to the ICRU reference volume to cover PTV, in the P only group, was 72 Gy. In the second group the prescribed dose to cover PTV1 was 66 Gy and to cover PTV2 was 6 Gy. In third group prescribed dose were 44 Gy for PTV1, 22 Gy for PTV2 and 6 Gy for PTV3.

All patients treated with definitive radiotherapy received 72 Gy in 36 fractions.

Patients who were irradiated after radical prostatectomy had one or two dose volume groups. If pN status was N0 they received 66 Gy to the PTV (prostate bed + /-SV). If pN status was Nx or N1 they received 44 Gy to PTV1 (cover pelvic lymph node and the prostate + /- SV bed) followed by 22 Gy to PTV2 (cover the prostate + /- SV bed). The SV bed was irradiated in patients with stage pT3b in the pathological report. All patients treated with postoperative or salvage radiotherapy received 66 Gy in 33 fractions.

### Determination of serum cytokine concentrations

The concentrations of IL-1β, IL-2, IL-6, IFN-γ and TGF-β1 in the sera of patients with prostate cancer were determined by commercial uncoated ELISA kits, according to manufacturer instructions (Invitrogen). The concentrations of cytokines were determined before radiotherapy, after 5th, 15th and 25th radiotherapy fractions, at the end of radiotherapy, and 1 month after the end of radiotherapy.

In brief, ELISA plates were coated with appropriate capture antibodies in coating buffer and incubated overnight at 4 °C. The plates were washed and blocked with ELISA/ELISASPOT Diluent. After 1 h incubation the plates were washed and serum samples and standards were added to the wells. The plates were incubated overnight at 4 °C for maximum sensitivity. After washing and aspiration, detection antibody solution was added to each well and plates were incubated for 1 h. Subsequently, the plates were washed and aspirated; avidin-HRP solution was added to the wells and incubated for 30 min. The plates were washed and (3,3′,5,5′-Tetramethylbenzidine) TMB solution was added to each well. The plates were incubated for 15 min at room temperature and the enzymatic reaction was terminated by adding stop solution. The absorbance was measured at a wavelength of 450 nm using Multiskan EX Thermo Labsystems plate reader. The serum samples were run in duplicate.

### Cytokine gene expression analysis

Cytokine gene expression in peripheral blood mononuclear cells (PBMCs) was examined at two time points: (1) at baseline (before radiotherapy) and (2) after the last fraction of radiotherapy. Total RNA from PBMCs was extracted with TRI Reagent (Sigma Aldrich). RNA was quantified on Biospec-nano (Shimadzu Biotech, Japan). Reverse transcription reaction preceded real-time quantitative PCR (RT-qPCR) reaction, performed with High Capacity cDNA Reverse Transcription Kit (Applied Biosystems by Thermo Fisher Scientific, Vilnius, Lithuania). The cDNA amplification reaction included primers against *INF-ɣ* (Hs00989291_m1), *IL-6* (Hs00174131_m1), and *TGF-β* (Hs00998133_m1) and Taqman2xUniversal PCR Master Mix, No Amperase UNG (Applied Biosystems, by Life Technologies, Warrington, UK). As endogenous control for data normalization, *GAPDH* (Hs02758991_g1) was used. Gene expression is presented in relative quantity (RQ) units using the comparative 2-ΔΔCt method on 7500 System SDS software (Applied Biosystems, Foster City, California, USA).

### Flow cytometry

Direct immunofluorescence staining was performed on peripheral blood (PB) using specific monoclonal antibodies (mAbs) and isotype-matched mAbs. Briefly, 50 μL of PB was incubated with 2.5 μL of appropriate monoclonal antibody combination for 15 min at room temperature in the dark. According to manufacturer’s protocol (Becton Dickinson), samples were lysed with 1X FACS Lysing Solution (Becton Dickinson), washed twice with phosphate-buffered saline (PBS) and fixed with 1% paraformaldehyde solution prior to acquisition. Surface marker expression was quantified on FACS Calibur flow cytometer (Becton Dickinson, San Jose, USA). A total of 10 000—50 000 gated events verified as peripheral blood lymphocytes (PBL), according to both, their physical characteristics forward scatter characteristics (FSC) and side scatter characteristics (SSC) were collected per sample and analyzed using CellQUEST software.

Within the lymphocyte gate we analyzed the percentage of CD3 (FITC-conjugated, Becton Dickinson R&D) and CD4 (PerCP-conjugated, Becton Dickinson) positive helper T cells and CD8 (PE-conjugated, Becton Dickinson), CD3 negative CD16CD56 positive (CD3-FITC-conjugated, CD16CD56-PE-conjugated Becton Dickinson) cells NK cells.

### Intracellular staining for Foxp3 transcription factor

Staining for Foxp3 transcription factor was performed using PE Mouse anti-human FoxP3 antibody and Human FoxP3 Buffer Set (BD Pharmingen). According to the manufacturer’s protocol. Briefly, the samples were stained for CD25 and CD4 cell surface markers, and washed with Stain Buffer (FBS) (BD Pharmingen). The cells were fixed with Human FoxP3 Buffer A and permeabilized with Human FoxP3 Buffer C. After resuspension in 100 μL of FBS Stain Buffer the cells were incubated with 5 µL of antibody specific for FoxP3 for 30 min at room temperature, protected from light and washed with Stain Buffer prior to flow cytometry analysis.

### Statistical analysis

Depending on the type of variables and distribution normality, data are presented as n (%), average ± SD or median (min–max). The relationship between variables was estimated using the Spearman coefficient of correlation. For the modeling of the relationship of the ordinal dependent variable (acute genitourinary toxicity) in repeated measurements with potential predictors, a multilevel ordinal regression was used. Changes in outcomes were examined across the study time course with a linear mixed effects modeling approach implemented in the lme4 package for the R statistical computing environment (R Core Team, Vienna, Austria,2019)^18^. Toxicity predictors from univariate analyzes which were statistically significant were included in the multivariate multilevel ordinal regression model. The level of statistical significance was set at 0.05.

The correlation between percentage of specific lymphocyte subsets and gene expression levels at pre-treatment time point and at the last fraction was analyzed using Spearman’s correlation.

## Supplementary information

Supplementary Information 1.

Supplementary Information 2.

## Data Availability

The datasets used and/or analysed during the current study are available from the corresponding author on reasonable request.
