# Supplementary material for: Evaluation of cytokine expression and circulating immune cell subsets as potential parameters of acute radiation toxicity in prostate cancer patients
Source: Sci Rep. 2020 Nov 4;10:19002. doi: 10.1038/s41598-020-75812-0 (PMC7643057; doi:10.1038/s41598-020-75812-0)
Supplement: Supplementary file 1 — Supplementary Information 1. [file 41598_2020_75812_MOESM1_ESM.docx]

**Supplementary Table 1.** Univariate analysis of serum cytokine levels in subgroups of patients with treated with definitive radiotherapy (d. RT) and patients treated with postoperative radiotherapy (p.RT)

|  | **Univariate analysis** | | | | | | | | |
| --- | --- | --- | --- | --- | --- | --- | --- | --- | --- |
|  | **Concentration**  **[pg/mL]** | **Pre-treatment** | **After 5^th^ fraction** | **After 15^th^ fraction** | **After 25^th^ fraction** | **After last fraction** | **After 1 month** | **b** | **p** |
| **Patients treated with d. RT** | **IL-1β** | 1.1 (0.1-4.1) | 1.0 (0.3-4.3) | 1.3 (0.3-4.2) | 1.2 (0.3-3.4) | 1.1 (0.3-4.3) | 1.5 (0.4-3.4) | 0.050 | 0.835 |
| **Patients treated with p. RT** |  | 1.4 (0.3-4.1) | 1.1 (0.2-3.1) | 1.3 (0.3-4.0) | 1.3 (0.4-4.3) | 1.2 (0.3-4.5) | 1.0 (0.2-4.8) |  |  |
| **Patients treated with d. RT** | **IL-2** | 1.8 (0.2-24.7) | 2.1 (0.2-17.8) | 1.6 (0.3-19.7) | 2.1 (0.4-9.8) | 1.7 (0.3-15.0) | 1.7 (0.5-18.8) | -0.038 | 0.976 |
| **Patients treated with p. RT** |  | 1.9 (0.6-27.9) | 1.7 (0.5-8.7) | 1.6 (0.4-32.2) | 1.9 (0.3-26.1) | 1.8 (0.2-36.4) | 1.8 (0.2-7.1) |  |  |
| **Patients treated with d. RT** | **IL-6** | 4.9 (0.8-14.3) | 4.6 (0.8-29.3) | 3.5 (0.6-40.0) | 4.8 (0.8-33.8) | 4.7 (0.5-58.6) | 5.2 (0.6-17.3) | 4.597 | **0.001** |
| **Patients treated with p. RT** |  | 1.3 (0.3-4.7) | 1.4 (0.2-6.2) | 1.7 (0.2-11.7) | 1.7 (0.2-8.4) | 1.6 (0.04-8.5) | 1.4 (0.1-12.3) |  |  |
| **Patients treated with d. RT** | **IFN-γ** | 9.3 (2.2-96.3) | 13.6 (1.3-52.1) | 10.7 (1.3-78.0) | 12.9 (1.8-90.5) | 11.9 (1.3-107.7) | 11.5 (1.9-52.9) | 8.869 | **0.020** |
| **Patients treated with p. RT** |  | 7.5 (3.2-29.2) | 7.7 (2.3-32.3) | 7.4 (2.7-30.9) | 8.3 (1.8-44.7) | 7.4 (1.3-36.1) | 5.1 (1.9-50.4) |  |  |
| **Patients treated with d. RT** | **TGF-β1** | 41.3 (6.4-155.0) | 43.1 (6.4-91.0) | 51.3 (5.7-149.7) | 38.5 (6.9-150.3) | 42.5 (6.9-89.4) | 32.0 (2.8-79.6) | -0.571 | 0.933 |
| **Patients treated with p. RT** |  | 43.4 (4.3-125.9) | 44.9 (4.2-70.4) | 43.7 (4.9-134.4) | 47.3 (5.9-120.1) | 51.6 (1.5-120.7) | 52.6 (3.3-193.5) |  |  |
