# Supplementary material for: Evaluation of cytokine expression and circulating immune cell subsets as potential parameters of acute radiation toxicity in prostate cancer patients
Source: Sci Rep. 2020 Nov 4;10:19002. doi: 10.1038/s41598-020-75812-0 (PMC7643057; doi:10.1038/s41598-020-75812-0)
Supplement: Supplementary file 2 — Supplementary Information 2. [file 41598_2020_75812_MOESM2_ESM.docx]

**Supplementary Table 2**. Univariate analysis of percentage of leukocyte/lymphocyte subsets in patients with prostate cancer during course of radiotherapy

| **Univariate analysis*** | | | | | | | | | |
| --- | --- | --- | --- | --- | --- | --- | --- | --- | --- |
| **Percentage of leukocyte/lymphocyte subset** | **Pre-treatment** | **After 5^th^ fraction** | **After 15^th^ fraction** | **After 25^th^ fraction** | **After last fraction** | **After 1 month** | **b** | **p** |  |
| **Lymphocytes** | 29.8±10.0 | 25.7±9.6 | 22.0±8.9 | 18.7±8.7 | 18.9±8.2 | 22.9±8.5 | -0.016 | **0.005** |  |
| **Monocytes** | 6.9±1.8 | 8.9±4.7 | 9.4±5.2 | 10.1±5.3 | 10.3±7.4 | 11.7±13.5 | 0.001 | 0.878 |  |
| **Granulocytes** | 54.0±9.4 | 55.5±13.2 | 58.1±11.6 | 59.5±11.9 | 59.4±12.6 | 57.0±12.1 | 0.008 | **0.038** |  |
| **Granulocytes/lymphocytes** | 2.0±0.8 | 2.5±1.2 | 3.2±1.8 | 4.0±2.5 | 3.8±2.0 | 2.9±1.2 | 0.113 | **<0.001** |  |
| **CD3^+^** | 70.6±13.7 | 69.4±13.9 | 70.3±12.9 | 68.4±14.3 | 71.3±13.1 | 68.2±15.0 | -0.001 | 0.750 |  |
| **CD3^+^4^+^** | 39.1±10.8 | 40.3±9.8 | 40.4±9.4 | 39.1±11.3 | 39.4±9.7 | 34.7±9.5 | 0.005 | 0.323 |  |
| **CD3^+^8^+^** | 29.9±12.9 | 29.2±11.6 | 29.3±12.0 | 28.5±11.2 | 32.2±13.1 | 33.9±13.1 | -0.001 | 0.792 |  |
| **CD3b^+^** | 69.5±13.5 | 67.7±14.7 | 67.7±14.9 | 68.1±14.5 | 68.8±12.9 | 66.4±15.8 | <0.001 | 0.954 |  |
| **NK** | 12.0 (2.3-34.9) | 12.0 (2.1-33.5) | 14.3 (1.7-56.3) | 14.4 (2.4-41.3) | 12.2 (3.9-36.3) | 15.2 (4.5-50.4) | -0.001 | 0.844 |  |
| **NKT** | 5.5 (0.8-34.7) | 5.6 (0.9-28.9) | 6.4 (0.8-30.9) | 8.2 (1.5-26.2) | 7.0 (1.6-27.8) | 8.3 (1.9-39.2) | 0.005 | 0.435 |  |
| **CD4^+^25^+^** | 1.0 (0.1-5.6) | 1.0 (0.1-2.8) | 1.0 (0.1-2.5) | 0.8 (0.2-3.8) | 0.9 (0.2-4.2) | 0.9 (0.2-2.6) | -0.037 | 0.549 |  |
| **CD4^+^CD25^+^ Foxp3 gated** | 13.8 (0.1-49.4) | 15.4 (1.0-58.1) | 12.6 (1.2-47.8) | 19.0 (0.1-63.4) | 14.9 (1.9-56.9) | 18.2 (0.9-62.4) | -0.004 | 0.166 |  |
| **CD4+ CD25+ Foxp3 mean**  **fluorescence intensity** | 26.6 (11.5-100.5) | 26.5 (9.6-85.7) | 22.8 (13.4-218.5) | 28.6 (6.0-72.1) | 32.7 (10.9-156.7) | 25.0 (13.5-77.9) | <0.001 | 0.989 |  |

*multilevel ordinal regression models with the degree of acute genitourinary toxicity as the dependent variable, adjusted for the time point and the type of treatment. Depending on the type of variables and the normality of distribution, data are shown as average ± standard deviation or median (min-max).
